# Recent Developments in Breast Cancer Diagnosis and Treatment

**DOI:** 10.3390/medicina62071260

**Published:** 2026-06-30

**Authors:** Jimmy Efird, Tithi Biswas

**Affiliations:** 1 VA Cooperative Studies Program Coordinating Center, 2 Avenue de Lafayette, Boston, MA 02111, USA; 2 Department of Radiation Oncology, School of Medicine, Case Western Reserve University, Cleveland, OH 44206, USA; tithi.biswas@ufhealth.org; 3 Department of Radiation Oncology, University of Florida, Gainesville, FL 32610, USA

The field of breast cancer diagnosis and treatment has experienced significant advances, leading to a decreasing mortality trajectory. An estimated 27 deaths per 100,000 resident U.S. population were reported in the year 2000, steadily declining to less than 10 by 2025 (Figure 1). However, the reduction is not uniform across demographic groups, with non-Hispanic Black females still dying at a rate of 26 per year. Geographic variability also exists in the age-adjusted annual death rate for breast cancer, with evident clusters occurring in the Southeast and lower Mississippi river regions (Figure 2). The differences in death rates likely reflect variable access and utilization to care, social economic position, genetics, environmental exposures, diet, exercise, mental health, and other medical and social determinants of health.

On the global front, breast cancer persists as a common cancer in women, with an estimated 670,000 deaths per year, but unequal distribution [1]. Among those living in a country at the upper end of the Human Development Index (HDI), 1 in 12 women will be diagnosed with this cancer during their lifetime, with a 1 in 71 mortality rate. On the other hand, in countries with a low HDI, 1 in 27 women will be diagnosed, with a 1 in 48 mortality rate. Like in the United States, early and timely diagnosis, effective interventions, and advances in research play a critical role in reducing breast cancer morbidity and mortality. Nonetheless, among women under the age of 50, a global increase of early-onset breast cancer has manifested as a prominent rise in cancer-related disability-adjusted life years [2].

Consisting of 20 papers published in *Medicina*, the Special Issue “Recent Developments in Breast Cancer Diagnosis and Treatment” focuses on reducing breast cancer risk through targeted screening, treatment advances, and novel research efforts [contributions 1–20]. Together, these manuscripts provide an informative overview of recent developments in the field with the aim of filling in knowledge gaps and stimulating future research. Overall, this information helps to establish a more refined risk profile for breast cancer, leading to better prevention, earlier detection, and superior treatment.

We wish to express our gratitude to the authors who have contributed to this Special Issue and hope that their insights and dedication in combatting breast cancer will help to save lives and enhance survivors’ quality of life. Breast cancer remains a perplexing challenge, requiring the deployment and scalability of multiple strategies to improve outcomes. Foremost, as illustrated in this collection of papers, is the integration of basic and medical sciences, community outreach, and culturally aligned education efforts. The systemic improvement of existing technologies, coupled with those on the emerging forefront of science, paints a bright future for breast cancer diagnosis and treatment.

As healthcare systems face a critical inflection point in terms of population growth and financial sustainability, the transformation of research into action, provider awareness, and stakeholder commitment will be pivotal as we advance into the future of this field. This will involve carefully targeting breast cancer resources while recognizing and overcoming structural barriers. Leveraging evidence-based approaches, predicated on sound science and trusted research, remains an important path forward.

## Figures and Tables

**Figure 1:**
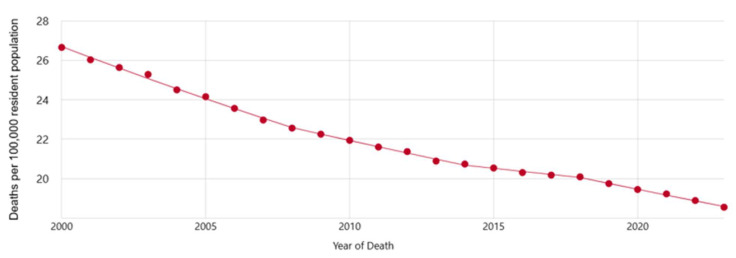
Breast cancer mortality by year of death among female residents (all ages) in the United States. Created by statecancerprofiles.cancer.gov on 27 May 2026.

**Figure 2:**
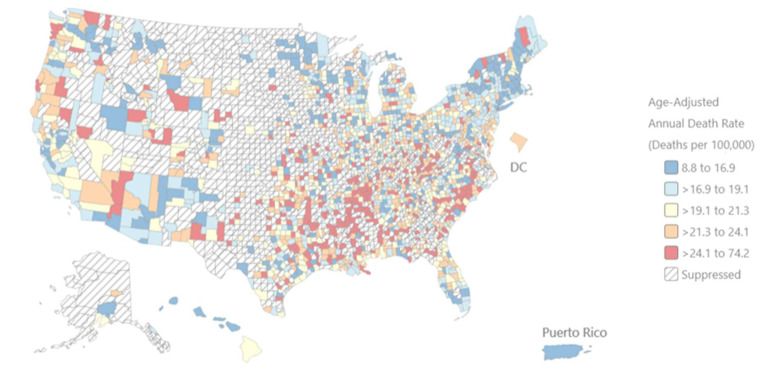
Age-adjusted annual breast cancer death rates (females, all ages) per 100,000 for the United States by county. Created by statecancerprofiles.cancer.gov on 27 May 2026.
